# Risk-stratified monitoring for sulfasalazine toxicity: prognostic model development and validation

**DOI:** 10.1136/rmdopen-2023-003980

**Published:** 2024-03-07

**Authors:** Abhishek Abhishek, Matthew Grainge, Tim Card, Hywel C Williams, Maarten W Taal, Guruprasad P Aithal, Christopher P Fox, Christian D Mallen, Matthew D Stevenson, Georgina Nakafero, Richard Riley

**Affiliations:** 1 Academic Rheumatology, University of Nottingham, Nottingham, UK; 2 Lifespan and Population Health, University of Nottingham, Nottingham, UK; 3 Centre for Evidence Based Dermatology, University of Nottingham, Nottingham, UK; 4 Translational Medical Sciences, University of Nottingham, Nottingham, UK; 5 Nottingham NIHR BRC, Nottingham, UK; 6 School of Meicine, Keele University, Keele, UK; 7 School of Medicine and Population Health, University of Sheffield, Sheffield, UK; 8 University of Birmingham, Birmingham, UK

**Keywords:** sulfasalazine, treatment, arthritis, rheumatoid, arthritis, psoriatic

## Abstract

**Background:**

Sulfasalazine-induced cytopenia, nephrotoxicity and hepatotoxicity is uncommon during long-term treatment. Some guidelines recommend 3 monthly monitoring blood tests indefinitely during long-term treatment while others recommend stopping monitoring after 1 year. To rationalise monitoring, we developed and validated a prognostic model for clinically significant blood, liver or kidney toxicity during established sulfasalazine treatment.

**Design:**

Retrospective cohort study.

**Setting:**

UK primary care. Data from Clinical Practice Research Datalink Gold and Aurum formed independent development and validation cohorts.

**Participants:**

Age ≥18 years, new diagnosis of an inflammatory condition and sulfasalazine prescription.

**Study period:**

1 January 2007 to 31 December 2019.

**Outcome:**

Sulfasalazine discontinuation with abnormal monitoring blood-test result.

**Analysis:**

Patients were followed up from 6 months after first primary care prescription to the earliest of outcome, drug discontinuation, death, 5 years or 31 December 2019. Penalised Cox regression was performed to develop the risk equation. Multiple imputation handled missing predictor data. Model performance was assessed in terms of calibration and discrimination.

**Results:**

8936 participants were included in the development cohort (473 events, 23 299 person-years) and 5203 participants were included in the validation cohort (280 events, 12 867 person-years). Nine candidate predictors were included. The optimism adjusted R^2^_D_ and Royston D statistic in the development data were 0.13 and 0.79, respectively. The calibration slope (95% CI) and Royston D statistic (95% CI) in validation cohort was 1.19 (0.96 to 1.43) and 0.87 (0.67 to 1.07), respectively.

**Conclusion:**

This prognostic model for sulfasalazine toxicity uses readily available data and should be used to risk-stratify blood-test monitoring during established sulfasalazine treatment.

**WHAT IS ALREADY KNOWN ON THIS TOPIC**

- Hepatic, haematological and renal toxicity from sulfasalazine occurs uncommonly after the first few months of treatment.
- Nevertheless, the manufacturers and some specialist societies, for example, the American College of Rheumatology recommend monitoring blood tests at 3 monthly intervals during established treatment.
- Other guidelines, for example, from the British Society of Rheumatology recommend no monitoring after the first 2 years of treatment.
- It is not known whether hepatic, haematological and renal toxicities due to sulfasalazine can be predicted and monitoring be risk-stratified.

**WHAT THIS STUDY ADDS**

- This study developed a prognostic model that discriminated patients at varying risk of sulfasalazine toxicity during long-term treatment.
- It had excellent performance characteristics in an independent validation cohort.
- The model performed well across age groups, and in people with rheumatoid arthritis and other inflammatory conditions.
- Any cytopenia or liver enzyme elevation prior to start of follow-up, chronic kidney disease stage 3, diabetes, methotrexate prescription, leflunomide prescription and age were strong predictors of sulfasalazine toxicity.

**HOW THIS STUDY MIGHT AFFECT RESEARCH, PRACTICE OR POLICY**

- This prognostic model uses information that can be easily ascertained during clinical visits.
- It can be used to inform decisions on the interval between monitoring blood tests.
- The results of this study ought to be considered by national and international rheumatology guideline writing groups to rationalise monitoring during long-term sulfasalazine treatment.

## Introduction

Sulfasalazine is commonly used in the treatment of inflammatory diseases such as rheumatoid arthritis (RA), psoriatic arthritis (PsA), axial spondylarthritis, reactive arthritis and infrequently in the management of inflammatory bowel disease (IBD) (the latter is mostly treated with 5-aminosalicylates due to a better safety profile).^1–3^ Although effective, sulfasalazine can cause cytopenia and elevated liver enzymes typically in the first 3–6 months of treatment, although late-onset toxicity is reported.^4–16^ Sulfasalazine can also cause crystalluria and interstitial nephritis, and is not recommended in those with severe renal impairment.^17^ Cautious use is recommended in those with mild-to-moderate renal impairment.^17^

There is considerable inconsistency in guidance on how to monitor patients on long-term sulfasalazine treatment for asymptomatic bone marrow, liver and/or renal toxicity. The British Society of Rheumatology (BSR) guidelines recommend 2–4 weekly blood tests for full blood count (FBC), liver function test (LFT), urea electrolytes and creatinine (UE&C) for the first 3 months of treatment followed by 3 monthly testing in the first year and no further monitoring blood tests thereafter.^18^ On the contrary, the American College of Rheumatology (ACR) guidelines recommend close monitoring for the first 3 months of treatment, followed by 3 monthly blood testing for FBC, UE&C and LFT during the entire duration of treatment.^19^ The summary of product characteristics for sulfasalazine recommends monitoring with FBC, LFT and UE&C at 3 monthly intervals during long-term treatment.^20^ However, whether everyone needs a fixed monitoring schedule once established on sulfasalazine treatment, or whether monitoring can be risk-stratified during long-term treatment is not known.

To predict clinically significant laboratory abnormalities during established sulfasalazine treatment and to inform the frequency of testing, we have developed and validated a prognostic model for clinically significant myelotoxicity, hepatotoxicity and/or nephrotoxicity due to sulfasalazine.

## Methods

### Data source

Data from the Clinical Practice Research Datalink (CPRD) Aurum and Gold were used for model development and validation, respectively.^21 22^ CPRD is an anonymised longitudinal database of electronic health records originated during clinical care in the National Health Service in the UK. With almost universal coverage of UK residents, participants that contributed data to the CPRD are representative of the UK population.^21^ The CPRD includes information on demographic details, lifestyle factors (eg, smoking, alcohol intake), diagnoses, results of blood tests and details of primary care prescriptions. CPRD Gold and Aurum complement each other in terms of coverage of general practices due to their use of different software for data capture. Some general practices that have contributed data to both databases are identifiable using a bridging file provided by the CPRD.

### Study design

Retrospective cohort study.

### Study period

1 January 2007 to 31 December 2019.

### Study population

Participants aged 18 years or older with a new diagnosis of inflammatory disease (eg, RA, axial spondyloarthritis, PsA and IBD) and prescribed sulfasalazine by their general practitioner (GP) for ≥6 months were eligible. Patients were required to have ≥1 year disease-free registration in their current general practice to be classified as having a new diagnosis.^23^ Additionally, patients were required to have received their first sulfasalazine prescription either after the first record of inflammatory disease in the CPRD or in the 90 days preceding. This 90-day period was allowed because recording of diagnosis may lag prescriptions. These two requirements minimised the chance of patients on long-term sulfasalazine treatment appearing as new users of sulfasalazine when they moved to a different general practice. Patients with chronic liver disease, haematological disease and chronic kidney disease (CKD) stage 4 or 5 prior to cohort entry were excluded as described in a previous manuscript.^24^

### Sulfasalazine prescriptions

In the UK, sulfasalazine initiation and dose escalation occur in hospital outpatient clinics. During this period prescriptions are issued by the hospital specialists. They also organise monitoring blood tests and acts on any abnormalities. Once a patient is established on treatment, typically approximately 6 months after initiating on treatment, the responsibility for prescribing and monitoring, including with periodic blood tests is handed to the patients’ GP as per the National Health Service (NHS) shared-care protocols. During shared-care monitoring, the GP seeks advice from the hospital specialist if there are side effects including abnormal blood-test results, and treatment changes are directed by the specialist.

### Start of follow-up

Patients were followed up from 180 days after their first primary care sulfasalazine prescription until the earliest of outcome, death, transfer out of practice, 90-day prescription gap, last data collection from practice, 31 December 2019 or 5 years.

### Outcome

Sulfasalazine toxicity-associated drug discontinuation was the outcome of interest. This was defined as a prescription gap of ≥90 days with either an abnormal blood-test result or a diagnostic code for abnormal blood-test result within ±60 days of the last prescription date.^25^ The blood tests were considered abnormal if any of the following were present: total leucocyte count <3.5×10^9^/L, neutrophil count <1.6×10^9^/L, platelet count <140×10^9^/L, alanine transaminase (ALT) and/or aspartate transaminase (AST) >100 IU/mL and decline in kidney function, defined as either progression of CKD based on medical codes recorded by the GP or >26 µmol/L increase in creatinine concentration, the threshold for consideration of acute kidney injury (AKI).^18 26^ In a previous validation study on methotrexate discontinuation, only 5.4% of abnormal blood-test results in this time window were potentially explained by an alternate illness.^25^

A random sample of sulfasalazine discontinuation with abnormal blood-test results was drawn. Data for all diagnostic codes entered during primary care consultations within ±60 days of the abnormal blood test result were extracted. AA (rheumatology and general medicine expertise) screened the list to identify outcomes that could potentially be explained by an alternative condition or its treatment.

### Predictors

These were selected by the clinical members of the study team based on their clinical expertise and knowledge of the published literature. Age, sex, body mass index (BMI), alcohol intake and diabetes were included as they associate with drug-induced liver injury (DILI).^27 28^ Individual inflammatory diseases were considered separately because sulfasalazine toxicity is reported to be less common in people with IBD than in those with RA.^3^ CKD stage 3 was included as it reduces sulfasalazine clearance.^29^ Statins, carbamazepine, valproate and paracetamol were included as their use is associated with sulfasalazine toxicity as per the British National Formulary. Methotrexate, leflunomide, thiopurines were included as they can cause cytopenia, elevated liver enzymes and AKI. Either cytopenia (neutrophil count <2×10^9^/L, total leucocyte count <4×10^9^/L or platelet count <150×10^9^/L) or elevated transaminase (ALT and/or AST >35 IU/L) during the first 6 months of primary care prescription were included as they predicted cytopenia and/or transaminitis in other studies.^30 31^

The latest record of demographic and lifestyle factors, diseases recorded within 2 years prior to start of follow-up and latest primary care prescriptions within 6 months prior to start of follow-up were used to define predictors except for CKD stage 3 that was defined using both GP records and/or estimated glomerular filtration rate 30–59 mL/min. GPs typically review patients with long-term conditions annually. A 2-year look-back period was used to minimise the risk of missing data from those that did not attend in the previous 12-months.

### Patient and public involvement

Patient and public involvement members were involved in selecting and prioritising the research question. They advised to use readily available datasets for the study rather than conduct an expensive and time-consuming clinical trial.

### Sample size

In a previously published cohort of 1321 patients with RA, 85 stopped sulfasalazine with neutropenia, thrombocytopenia or elevated liver enzymes during a mean follow-up of 2.39 years.^16^ Assuming a similar incidence of treatment discontinuation for model development, the minimum sample size needed to minimise model overfitting (a target shrinkage factor of 0.9) and ensure precise estimation of overall risk was 1748 participants (113 outcomes) based on a maximum of 25 parameters, Cox-Snell R^2^ value of 0.12, outcome rate of 0.027/person-year,^16^ a 5-year time horizon and a mean follow-up period of 2.39 years using the formulae of Riley *et al*.^32^ The sample size for external model validation was much larger than the typically recommended minimum sample size of 200 events.^33^

### Statistical analysis

Multiple imputation handled missing data on BMI, alcohol intake and sulfasalazine dose using chained equations.^34^ We carried out 10 imputations in the development dataset and 5 imputations in the validation dataset—a pragmatic approach considering the larger size of CPRD Aurum. The imputation model included all candidate predictors, Nelson-Aalen cumulative hazard function and outcome variable. The data analysis was undertaken using the Stata command ‘mi estimate’ in a combined dataset that included all imputations.

### Model development

Fraction polynomial regression (first-degree) analysis was used to model non-linear risk relationships with continuous predictors, but these were not better than the linear terms (p>0.05), hence were not transformed. All 12 candidate predictors (19 parameters) were included in the Cox model and coefficients of each parameter estimated and combined using Rubin’s rule across the imputed datasets. The risk equation for predicting an individual’s risk of sulfasalazine discontinuation with abnormal blood-test results by 5 years follow-up was formulated in the development data. The baseline survival function at *t=5 years*, a non-parametric estimate of survival function when all predictor values are set to zero, which is equivalent to the Kaplan-Meier product-limit estimate, was estimated along with the estimated regression coefficients (β) and the individual’s predictor values (X). This led to the equation for the predicted absolute risk over time^35^:

Predicted risk of sulfasalazine toxicity associated drug discontinuation at 5 years=1–S_0_(t=5)^exp(βX)^, where S_0_(t=5) is the baseline survival function at 5 years of follow-up and βX is the linear predictor, β_1_x_1_+β_2_x_2_+ … + β_p_x_p_.

### Model internal validation and shrinkage

The performance of the model in terms of calibration (where 1.00 is the ideal) was assessed by plotting agreement between predicted and observed outcomes. Internal validation was performed to correct performance estimates for optimism due to overfitting by bootstrapping with replacement of 500 samples of the development data. The full model was fitted in each bootstrap sample and then its performance was quantified in the bootstrap sample (apparent performance) and the original sample (test model performance), and the optimism calculated (difference in test performance and apparent performance). A uniform shrinkage factor was estimated as the average of calibration slopes from the bootstrap samples. This process was repeated for all 10 imputed datasets, and the final uniform shrinkage calculated by averaging across the estimated shrinkage estimates from each imputation. Optimism-adjusted estimates of performance for the original model were then calculated, as the original apparent performance minus the optimism.

To account for overfitting during model development process, the original β-coefficients were multiplied by the final uniform shrinkage factor and the baseline hazards re-estimated conditional on the shrunken β-coefficients to ensure that overall calibration was maintained, producing a final model. The D statistic, a measure of discrimination, interpreted as a log HR, the exponential of which gives the HR comparing two groups defined by above/below the median of the linear predictor was calculated.^36 37^ R^2^, a measure of variation explained by the model was calculated.

### Model external validation

External validation of the final model was performed using data from CPRD Gold. The final developed model equation was applied to the validation dataset, and calibration and discrimination were examined using the same measures as above.^36 37^ Calibration of 5-year risk was examined by plotting agreement between estimated risk from the model and observed outcome risks. In the calibration plot, predicted and observed risks were divided into 10 equally sized groups. Additionally, pseudo-observations were used to construct smooth calibration curves across all individuals via a running non-parametric smoother. Separate graphs were plotted for each imputation of the validation cohort and an example of one plot is shown in the results. Subgroup analyses considered age group and inflammatory disease type (RA vs others). Stata-MP V.16 was used for all statistical analyses. This study was reported in line with the transparent reporting of a multivariate prediction model for individual prediction or diagnosis guidelines.^38^

## Results

### Study participants

Data for 8936 and 5203 participants contributing 23 299 and 12 867 person-years follow-up were included in the derivation and validation cohorts, respectively (online supplemental figures S1 and S2). Most participants in both cohorts were diagnosed with RA, were female and had similar prevalence of lifestyle factors, comorbidities and drug treatments (table 1). Nine candidate predictors (21 parameters) were included in the model (table 2).

10.1136/rmdopen-2023-003980.supp1 Supplementary data

**Table 1:** Distribution of candidate predictors in development and validation cohorts

Predictor*	Development cohort (CPRD Aurum) N=8936	Validation cohort (CPRD Gold) N=5203
Age, mean (SD) year	55.3 (14.8)	55.5 (14.8)
Female sex	5535 (61.9)	3240 (62.3)
Body mass index (kg/m^2^)		
<18.5	138 (1.5)	88 (1.7)
18.5–24.9	2441 (27.3)	1428 (27.5)
25.0–29.9	2840 (31.8)	1678 (32.3)
≥30	2714 (30.4)	1626 (31.3)
Missing	803 (9.0)	383 (7.4)
Alcohol use		
Non-user	1705 (19.1)	805 (15.5)
Low (1–14 units/week)	3854 (43.1)	2859 (55.0)
Moderate (15–21 units/week)	535 (6.0)	251 (4.8)
Hazardous (>21 units/week)	667 (7.5)	273 (5.3)
Ex-user	996 (11.2)	359 ((6.9)
Missing	1179 (13.2)	656 (12.6)
Inflammatory conditions		
Rheumatoid arthritis	6945 (77.7)	4067 (78.2)
Psoriatic arthritis	1354 (15.2)	773 (14.9)
Inflammatory bowel disease	319 (3.6)	173 (3.3)
Ankylosing spondylitis/reactive arthritis	318 (3.6)	190 (3.7)
Comorbidities		
Diabetes	982 (11.0)	519 (10.0)
Chronic kidney disease stage 3	613 (6.9)	333 (6.4)
Immunosuppressive drugs		
Methotrexate	2999 (33.6)	1785 (34.3)
Leflunomide	109 (1.2)	78 (1.5)
Azathioprine/Mercaptopurine	73 (0.8)	41 (0.8)
Other drugs		
Statins	2088 (23.4)	1130 (21.7)
Carbamazepine/Valproate	103 (1.2)	37 (0.7)
Paracetamol	1445 (16.2)	884 (17.0)
At least mild cytopenia or liver enzyme elevation in 6 months preceding start of follow-up	1264 (14.2)	753 (14.5)

*Values are numbers (percentage) unless stated otherwise.

CPRD, Clinical Practice Research Datalink.

**Table 2:** Final model HRs and β-coefficients

	Adjusted HR (95% CI)	β-Coefficients
Age, mean (SD) year	1.01 (1.00 to 1.02)	0.0076439
Female sex	1.08 (0.88 to 1.31)	0.0741336
Body mass index	0.98 (0.97 to 1.00)	−0.0168035
Alcohol use		
Non-user	Reference	
Low (1–14 units/week)	1.02 (0.80 to 1.29)	0.0182851
Moderate (15–21 units/week)	0.64 (0.38 to 1.06)	−0.4507257
Hazardous (>21 units/week)	0.87 (0.58 to 1.33)	−0.133557
Ex-user	0.94 (0.67 to 1.32)	−0.0651469
Inflammatory conditions		
Rheumatoid arthritis	Reference	
Psoriatic arthritis	1.03 (0.78 to 1.36)	0.0316689
Inflammatory bowel disease	0.74 (0.38 to 1.44)	−0.305206
Ankylosing spondylitis/reactive arthritis	1.25 (0.74 to 2.12)	0.2214547
Comorbidities		
Diabetes	1.34 (1.01 to 1.78)	0.2909969
Chronic kidney disease stage 3	1.96 (1.47 to 2.62)	0.671859
Immunosuppressive drugs		
Methotrexate	1.39 (1.15 to 1.68)	0.3315573
Leflunomide	2.05 (1.09 to 3.86)	0.7164324
Azathioprine/Mercaptopurine	1.24 (0.37 to 4.17)	0.2189764
Other drugs		
Statins	0.98 (0.78 to 1.24)	−0.0181917
Carbamazepine/Valproate	0.74 (0.28 to 2.00)	−0.2949835
Paracetamol	1.14 (0.90 to 1.43)	0.1272515
Blood-test abnormalities		
At least mild cytopenia or liver enzyme elevation in 6 months preceding start of follow-up	2.80 (2.29 to 3.42)	1.029245

*The reported values are before shrinkage.

### Model development

In the derivation dataset, 473 outcome events occurred during the follow-up period at a rate (95% CI) of 20.30 (18.55 to 22.22) per 1000 person-years. Of these, 256, 131 and 113 patients, respectively stopped treatment due to cytopenia, renal function decline and elevated liver enzymes. Outcome validation exercise in 178 outcomes revealed that only 4.5% outcomes (n=8) could potentially be explained by another contemporaneous illness or its treatments, with a positive predictive value of 95.5% (online supplemental table S1).

Outcome events occurred throughout 5-year follow-up period when the entire cohort was considered (online supplemental figure S3) and when patients co-prescribed either methotrexate or leflunomide or thiopurine with sulfasalazine were excluded (online supplemental figure S4). CKD stage 3, diabetes (either type 1 or 2), co-prescription of methotrexate, co-prescription of leflunomide and either cytopenia or elevated liver enzymes during first 6 months of sulfasalazine prescription were strong predictors of drug discontinuation with adjusted HR (95% CI) 1.96 (1.47 to 2.62), 1.34 (1.01 to 1.78), 1.39 (1.15 to 1.68), 2.05 (1.09 to 3.86) and 2.80 (2.29 to 3.42), respectively (table 2). From the bootstrap, a uniform shrinkage factor of 0.84 was obtained and used to shrink predictor coefficients in the final model for optimism and after re-estimation, the final model’s cumulative baseline survival function (S_0_) was 0.940 at 5 years of follow-up (Box 1).

Box 1 **Equation to predict the risk of sulfasalazine discontinuation after 6 months of primary care prescription and within the next 5 years**

Risk score=1–0.940^exp(0.84βX)^, where βX=0.0076439×age in years at first primary care prescription+0.0741336×female sex−0.0168035×BMI+0.0182851×low alcohol intake−0.4507257×moderate alcohol intake−0.1335573×hazardous alcohol intake−0.0651469×e­x-alcohol intake+0.0316689×psoriasis−0.305206×IBD+0.2214547×­ankylosing spondylitis/reactive arthritis+0.2909969×diabetes+­0.671859×CKD+0.3315573×MTX+0.7164324×LEF+0.2189764×­AZA or 6-MP−0.0181917×statins−0.2949835×carbamazepine/­valproate+0.1272515×paracetamol+1.029245×at least mild cytopenia or liver enzyme elevation within 6 months of primary care sulfasalazine prescription.

All variables are code 0, and 1 if absent or present, respectively, except for BMI and age that were continuous variables. At 5 years, 0.940 is the baseline survival function, 0.84 is the shrinkage factor and the other numbers are the estimated regression coefficients for the predictors, which indicate their mutually adjusted relative contribution to the outcome risk.AZA, azathioprine; BMI, body mass index; CKD, chronic kidney disease; IBD, inflammatory bowel disease; LEF, leflunomide; MP, mercaptopurine; MTX, methotrexate.

### Model performance in the development cohort

**Figure 1:**
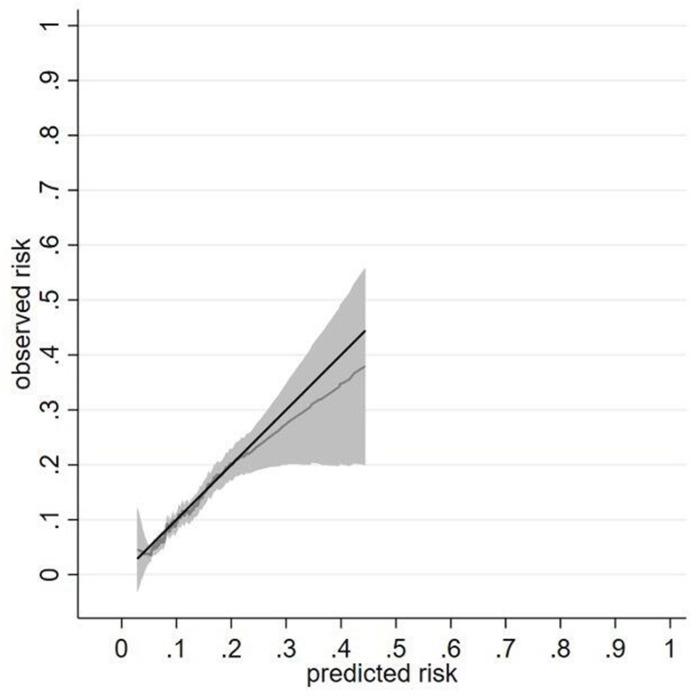
Calibration of a prognostic model for sulfasalazine discontinuation with abnormal monitoring blood-test results at 5 years in the development cohort. Data from a single imputed dataset was used; S_0_(t=5) 0.940.

As expected, the calibration slope (95% CI) in the development data was 1.00 (0.85 to 1.15). Calibration plot of the final (ie, after shrinkage) model at 5 years showed that the average model predictions matched the average observed outcome probabilities across 10 groups of patients, with CIs overlapping the 45-degree line (perfect prediction line) (figure 1). As most patients had a low risk of outcome (online supplemental figure S5), most of the deciles clustered at the bottom left of the calibration plot (online supplemental figure S6). The smoothed calibration curve at 5 years showed alignment of observed risk to the predicted risk with wide CIs at high-risk probabilities (figure 1). The Royston D statistic was 0.91 (95% CI 0.77 to 1.05), corresponding to an HR (95% CI) of 2.48 (2.16 to 2.86) comparing the risk of participants who were above the median of linear predictor to that below the median. The optimism adjusted Royston D statistic was 0.79, corresponding to an HR of 2.20 (table 3).

**Table 3:** Model diagnostics

Measure	Apparent performance*	Test performance†	Average optimism‡	Optimism corrected performance§	External validation (CPRD Aurum)¶
Overall calibration slope	1.00 (0.85 to 1.15)	0.84 (0.70 to 0.98)	0.16	0.84 (0.69 to 0.99)	1.19 (0.96 to 1.43)
R^2^_D_	0.17 (0.12 to 0.21)	0.15 (0.11 to 0.19)	0.04	0.13 (0.08 to 0.17)	0.15 (0.10 to 0.21)
Royston D statistic	0.91 (0.77 to 1.05)	0.85 (0.72 to 0.99)	0.12	0.79 (0.65 to 0.93)	0.87 (0.67 to 1.07)

*Refers to performance (95% CI) estimated directly from the data that were used to develop the model.

†Determined by executing full model in each bootstrap sample (500 samples with replacement), calculating bootstrap performance and applying same model in original sample.

‡Average difference between model performance in bootstrap data and test performance in original dataset.

§Subtracting average optimism from apparent performance.

¶Penalised model was externally validated (penalised calibration slope: 1.19; 95% CI 1.01 to 1.37).

CPRD, Clinical Practice Research Datalink.

### Model performance in the validation cohort

**Figure 2:**
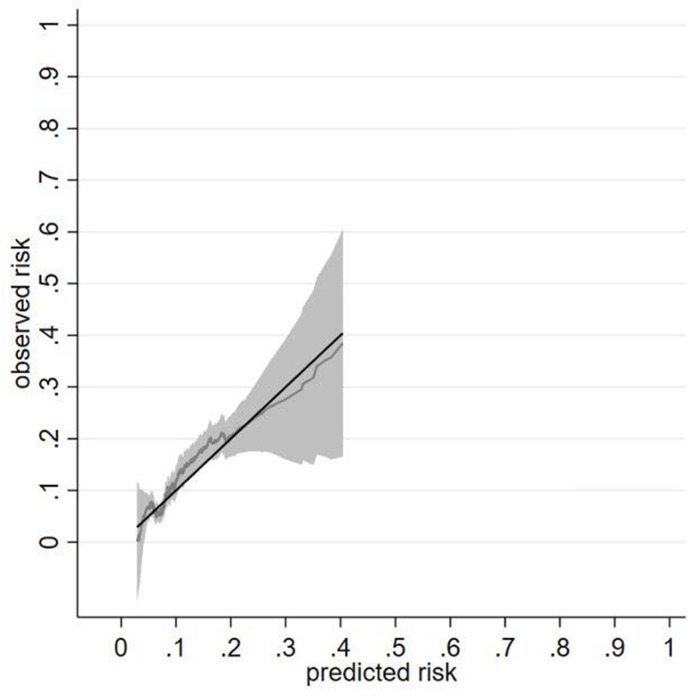
Calibration of a prognostic model for sulfasalazine discontinuation with abnormal monitoring blood-test results at 5 years in the validation cohort. Data from a single imputed dataset was used; S_0_(t=5) 0.940.

There were 280 outcomes at a rate (95% CI) of 21.76 (19.36 to 24.47)/1000 person-years in the validation cohort. The calibration slope (95% CI) across the 5-year follow-up period was 1.19 (0.96 to 1.43) (figure 2). The calibration plot showed reasonable correspondence between observed and predicted risk at 5 years across the tenths of risk (online supplemental figure S7). Most of the deciles clustered at the bottom left of the calibration plot due to a low risk of outcome for most patients (online supplemental figures S7 and S8). When individual risks were plotted, the smoothed calibration curve showed alignment of the predicted risk to the observed risk at low risk and wide CIs overlapping the perfect prediction line at high-risk probabilities (figure 2). Model performance was also tested at years 1, 2, 3 and 4 (online supplemental figures S9–S12) and showed a similar pattern except for overprediction of risk at 1 year. The Royston D statistic in the validation data was 0.87 (0.67 to 1.07), corresponding to an HR (95% CI) of 2.39 (1.95 to 2.92). Model discrimination in the derivation and validation data was broadly similar (table 3). The model performed well in those younger or older than 60 years, in those with RA or other conditions (online supplemental figures S13 and S14).

Worked examples: ten anonymised patient profiles, one from the middle of each of the 10 groups defined by deciles of predicted risk were selected from the development cohort, the higher the decile group the higher the risk, and the risk equation was applied to each. The cumulative probability of outcome over 5 years ranged from 5.3% in the middle of the first group to 9.3% in the middle of the seventh group, and 19.0% in the middle of the 10th group (online supplemental table S2).

## Discussion

We have developed and externally validated a prognostic model for sulfasalazine discontinuation due to abnormal blood-test results. To the best of our knowledge, this is the first such risk-prediction model. It performed well in predicting outcomes by 5 years and in clinically relevant subgroups defined by age and inflammatory condition. Previous studies have variably reported N-acetyltransferase 2 (NAT-2) acetylator status to be associated with sulfasalazine toxicity.^15 39^ However, these studies evaluated all side effects and did not separately assess either myelotoxicity, hepatotoxicity or nephrotoxicity as evaluated in the current study.

Our findings suggest that a one-size-fits-all approach to monitoring for blood, liver or renal toxicity using 3 monthly blood tests during long-term sulfasalazine treatment as recommended in the summary of product characteristics and the ACR guidelines, and not monitoring for these after the first year of treatment as recommended in the BSR guidelines are both inappropriate because there is a large interindividual variation in the risk of developing these side effects. The large variation in risk implies that it may be reasonable to not monitor some patients after the first year of sulfasalazine treatment, while others at higher risk of side effects are monitored frequently, for example, 3 monthly.

It is important to realise that DILI can be idiosyncratic and annual testing is unlikely to detect them early enough to improve patient outcome. It is beyond our remit to propose threshold at which the frequency of monitoring blood tests should be altered. These decisions are best taken by guideline writing groups. Thus, our findings ought to be considered by guideline writing groups.

It is important that the results of this study are not used to risk-stratify monitoring in patients newly started on sulfasalazine because our prognosis model used data from patients prescribed sulfasalazine by their GP for 6 months after initiating treatment and dose-escalation in a hospital outpatient. It typically takes 3–6 months to stabilise a patient’s sulfasalazine dose before prescription and monitoring is handed over to the GP. In healthcare systems where such shared care arrangements do not exist, this strategy may be applied after 1 year of sulfasalazine treatment. Although generally perceived to be safe, sulfasalazine use carries a risk of myelotoxicity and nephrotoxicity comparable to that observed with methotrexate in people with RA.^40^

CKD stage 3, diabetes and concomitant methotrexate or leflunomide therapy were associated with sulfasalazine discontinuation with abnormal monitoring blood-test results in this study. These associations may be due to reduced sulfasalazine clearance in CKD and DILI being associated with diabetes.^41^ Abnormal blood-test results during the first 6 months of therapy were associated with discontinuing sulfasalazine with abnormal monitoring blood-test results, like findings for methotrexate and leflunomide.^24 42^ Elevated liver enzymes and cytopenia before starting treatment have previously been associated with abnormal blood-test results in patients treated with methotrexate and biologics, respectively.^31 43–48^

There are several strengths of this study. First, we used a large real-world and nationally representative dataset for model development and a similar independent dataset for external validation. Second, the study population included patients with a range of diseases and the results have broad generalisability. Third, the prognostic factors were selected by an expert multidisciplinary team based on clinical experience. Fourth, our outcome required the abnormal blood-test result to be associated with sulfasalazine discontinuation, thus, allowing the model to predict clinically relevant outcomes. Fifth, the prognostic model is easy to use in practice, and can be easily built into GP electronic health records.

However, several limitations of this study ought to be considered. First, we did not have access to the date when the patient was first prescribed sulfasalazine in the hospital clinic. Second, we did not have data on concurrent use of biologics as these are hospital prescribed. However, there is no evidence to suggest that biologics increase sulfasalazine toxicity. Third, we did not have data on disease activity as these are not recorded in the CPRD. Fourth, the abnormal blood test could be due to a different illness and not due to sulfasalazine. However, only 4.5% of the outcomes in the development cohort in this study could be potentially explained by an alternate illness in this study. Similarly, in our previous validation studies on methotrexate, only 5.4% of abnormal blood-test results could be explained by an alternative illness.^25^ Fifth, although the external validation dataset was distinct from the model development dataset, it also originated from UK general practice. We recommend therefore that our model be validated in a dataset from another country. Sixth, there were 31 (0.3%) patients in the highest three risk groups defined according to tenths of risk, resulting in uncertainty regarding predictors for these groups. Seventh, we did not perform competing risk regression. However, this does not limit the validity of our findings as there were few deaths (28 (0.3%)) in the derivation cohort and 8 (0.2%) deaths in validation cohort up to 5-year follow-up period. Finally, this was a retrospective analysis using secondary data originated during routine care of patients in the NHS and data were not prospectively collected for this study. However, any bias from this approach was minimised by inclusion of all consecutive patients that were prescribed sulfasalazine within the study period that met the eligibility criteria.

In conclusion, we have developed and externally validated a prognostic model for sulfasalazine discontinuation with abnormal monitoring blood-test results. These findings need to be considered by national and international specialist societies’ guideline writing groups to decide on risk-stratified frequency of monitoring blood tests during long-term sulfasalazine treatment.

## Data Availability

Data may be obtained from a third party and are not publicly available. Data used in this study cannot be shared due to CPRD licensing requirements. However, CPRD data may be obtained directly from them.
