# Supplementary material for: Risk-stratified monitoring for sulfasalazine toxicity: prognostic model development and validation
Source: RMD Open. 2024 Mar 7;10(1):e003980. doi: 10.1136/rmdopen-2023-003980 (PMC10921482; doi:10.1136/rmdopen-2023-003980)

Contents

Table S1 Other conditions potentially explaining blood test abnormalities in patients discontinuing sulfasalazine treatment due to abnormal blood test results 2

Table S2 Individual patient's characteristics at the midpoint of each decile of risk 3

Figure S1: Study population selection criteria for model development 4

Figure S2: Model validation cohort: Study population selection criteria 5

Figure S3: Occurrence of cytopenia, myelotoxicity and renal function decline in sulfasalazine prescribed cohort 6

Figure S4: Occurrence of cytopenia, myelotoxicity and renal function decline in sulfasalazine prescribed cohort excluding patients prescribed either methotrexate, leflunomide, or thiopurines at cohort entry 7

Figure S5: Distribution of predicted risk in the model derivation cohort at 5 years 8

Figure S6: Calibration of a prognostic model for SSZ discontinuation with abnormal monitoring blood-test results at 5-years in the development cohort1 9

Figure S7: Calibration of a prognostic model for SSZ discontinuation with abnormal monitoring blood-test results at 5-years in the validation cohort1 10

Figure S8: Distribution of predicted risk in the model validation cohort at 5 years 11

Figure S9: Calibration of a prognostic model for SSZ discontinuation with abnormal monitoring blood-test results at 1 year in the validation cohort1 12

Figure S10: Calibration of a prognostic model for SSZ discontinuation with abnormal monitoring blood-test results at 2 years in the validation cohort1 13

Figure S11: Calibration of a prognostic model for SSZ discontinuation with abnormal monitoring blood-test results at 3 years in the validation cohort1 14

Figure S12: Calibration of a prognostic model for SSZ discontinuation with abnormal monitoring blood-test results at 4 years in the validation cohort1 15

Figure S13: Calibration of a prognostic model for SSZ discontinuation with abnormal monitoring blood-test results at 5 years in the validation cohort: stratified according to age. 16

Figure S14: Calibration of a prognostic model for SSZ discontinuation with abnormal monitoring blood-test results at 5 years in the validation cohort: stratified according to disease type. 17

Abhishek A, *et al. RMD Open* 2024; 10:e003980. doi: 10.1136/rmdopen-2023-003980

Table S1 Other conditions potentially explaining blood test abnormalities in patients discontinuing sulfasalazine treatment due to abnormal blood test results.

Participants	Read term
1	Cancer chemotherapy
1	Myelodysplasia
1	Fatty liver
1	Unspecified pyelonephritis NOS
1	Alcohol dependence syndrome
2	Acute pyelonephritis
1	Acute promyelocytic leukaemia

Table S2 Individual patient's characteristics at the midpoint of each decile of risk.

Decile	Age (yr.)	Sex	BMI (kg/m^2)	Alcohol	Disease	DM	CKD	Immune-suppress. drug	Statin	Anti-epileptic	Paracet amol	BTA	Cumulative probability of outcome at 5-years (%)
1	28	F	35.2	Moderate	PSA	No	No	MTX	No	No	No	No	5.25
2	37	M	23.0	Non-drinker	RA	No	No	No	No	No	No	No	6.19
3	29	F	21.6	Low	PSA	No	No	No	No	No	No	No	6.72
4	59	F	29.8	Low	RA	No	No	No	No	No	No	No	7.25
5	59	M	24.0	Non-drinker	RA	No	No	No	No	No	Yes	No	7.80
6	73	F	27.9	Low	RA	No	No	Aza/6-MP	No	Yes	Yes	No	8.45
7	48	F	25.3	Low	RA	No	No	MTX	No	No	No	No	9.29
8	79	M	27.7	Non-drinker	RA	Yes	No	No	Yes	No	Yes	No	10.52
9	69	M	30.6	Low	PSA	No	Yes	No	No	No	Yes	No	13.90
10	55	F	33.8	Non-drinker	RA	No	No	MTX	No	No	No	Yes	19.04

Aza/6-MP: - Azathioprine/6-Mercaptopurine; BMI: - Body Mass Index; BTA: - Blood Test abnormalities within 6 months of first primary care sulfasalazine prescription; CKD: - Chronic Kidney Disease; Anti-epileptics: - carbamazepine / valproate; DM: - diabetes mellitus; F: - female; M: - male; MTX: - methotrexate; PsA: - psoriatic arthritis; RA: - Rheumatoid Arthritis.

Abhishek A, *et al. RMD Open* 2024; 10:e003980. doi: 10.1136/rmdopen-2023-003980

Figure S1: Study population selection criteria for model development

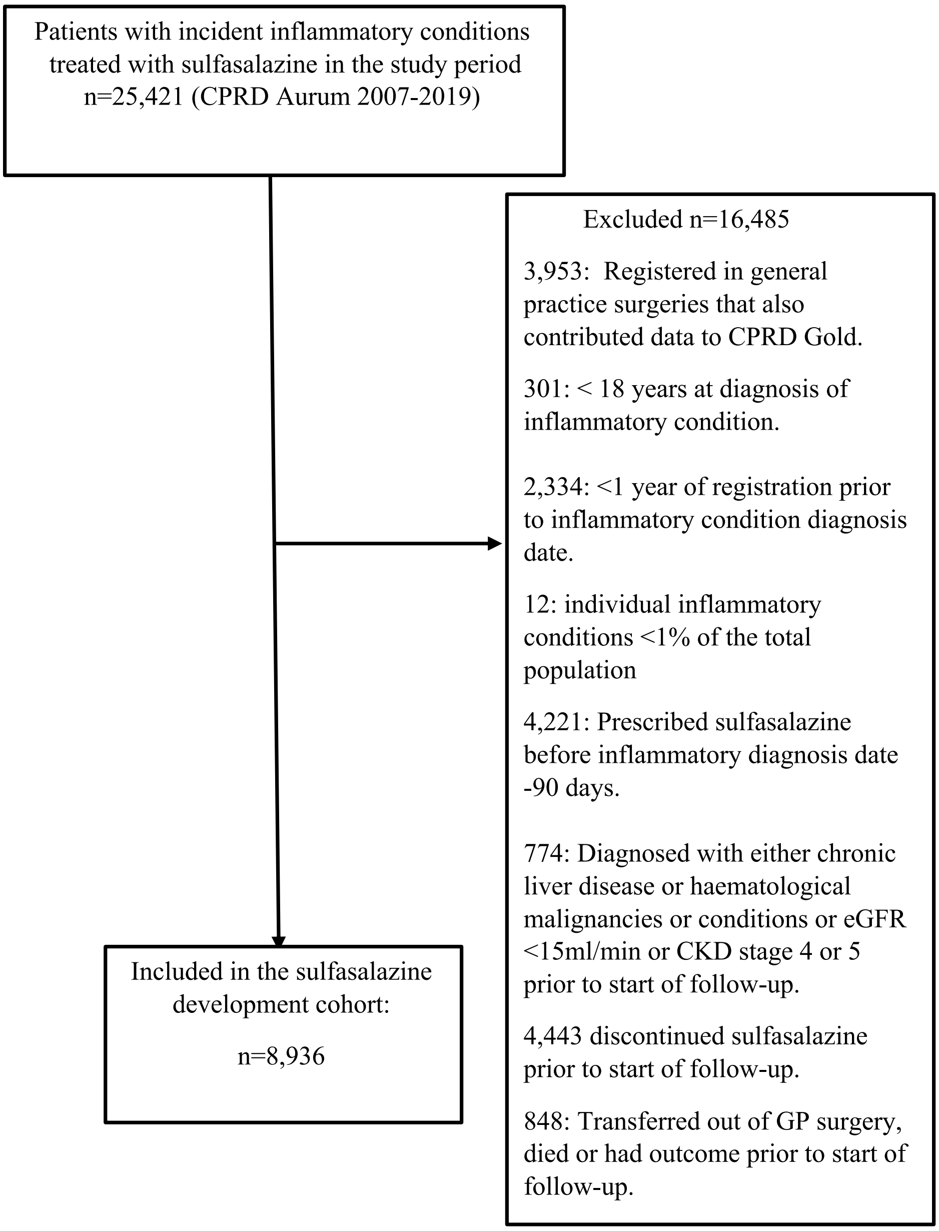

Figure S2: Model validation cohort: Study population selection criteria

Patients with incident inflammatory conditions treated with sulfasalazine in the study period n=12,775 (CPRD GOLD 2007-2019)

Excluded n=7,572

164: < 18 years at diagnosis of inflammatory condition.

1,010: <1 year of registration prior to AIRD diagnosis date.

8: individual inflammatory conditions <1% of the total population

2,699: Prescribed sulfasalazine before inflammatory condition diagnosis date -90 days.

243: Diagnosed with either chronic liver disease or haematological malignancies or conditions or eGFR <15ml/min or CKD stage 4 or 5 prior to start of follow-up

2,744: Discontinued sulfasalazine prior to start of follow-up.

702: Transferred out of GP surgery, died or had outcome prior to start of follow-up.

Included in the Sulfasalazine model validation cohort: n=5,203

Abhishek A, *et al. RMD Open* 2024; 10:e003980. doi: 10.1136/rmdopen-2023-003980

Figure S3: Occurrence of cytopenia, myelotoxicity and renal function decline in the cohort exposed to sulfasalazine

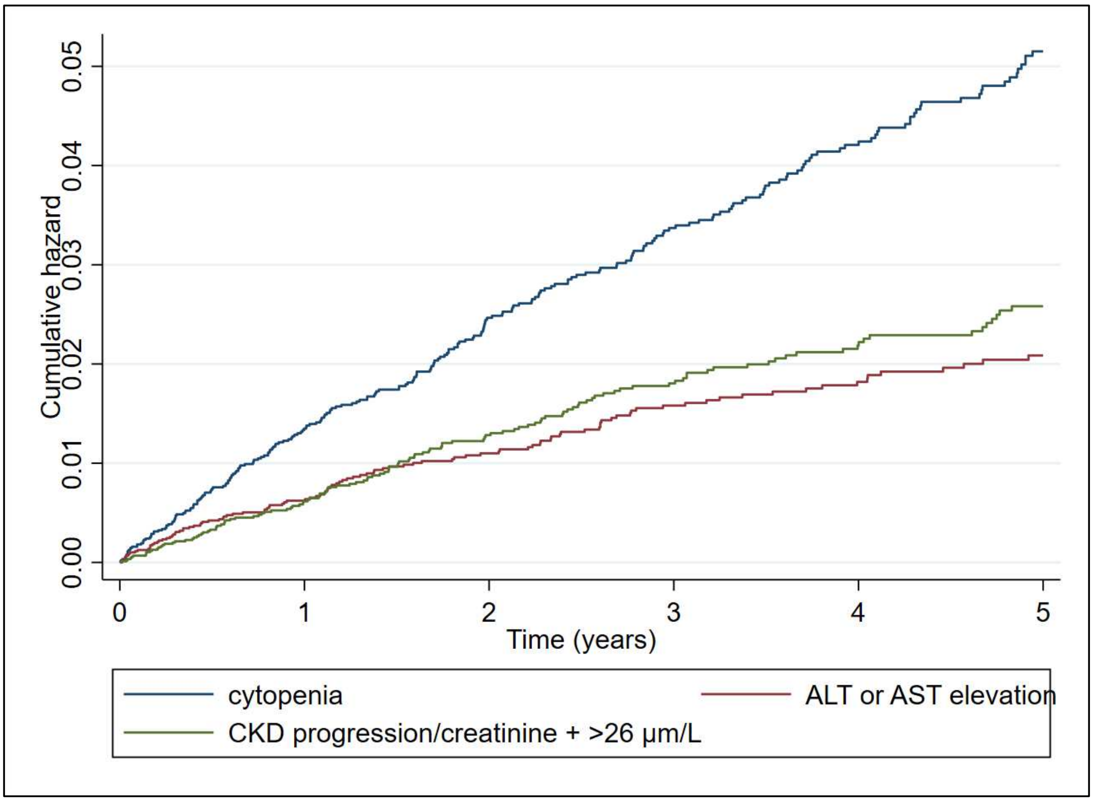

Figure S4: Occurrence of cytopenia, myelotoxicity and renal function decline in sulfasalazine prescribed cohort excluding patients prescribed either methotrexate, leflunomide, or thiopurines at cohort entry

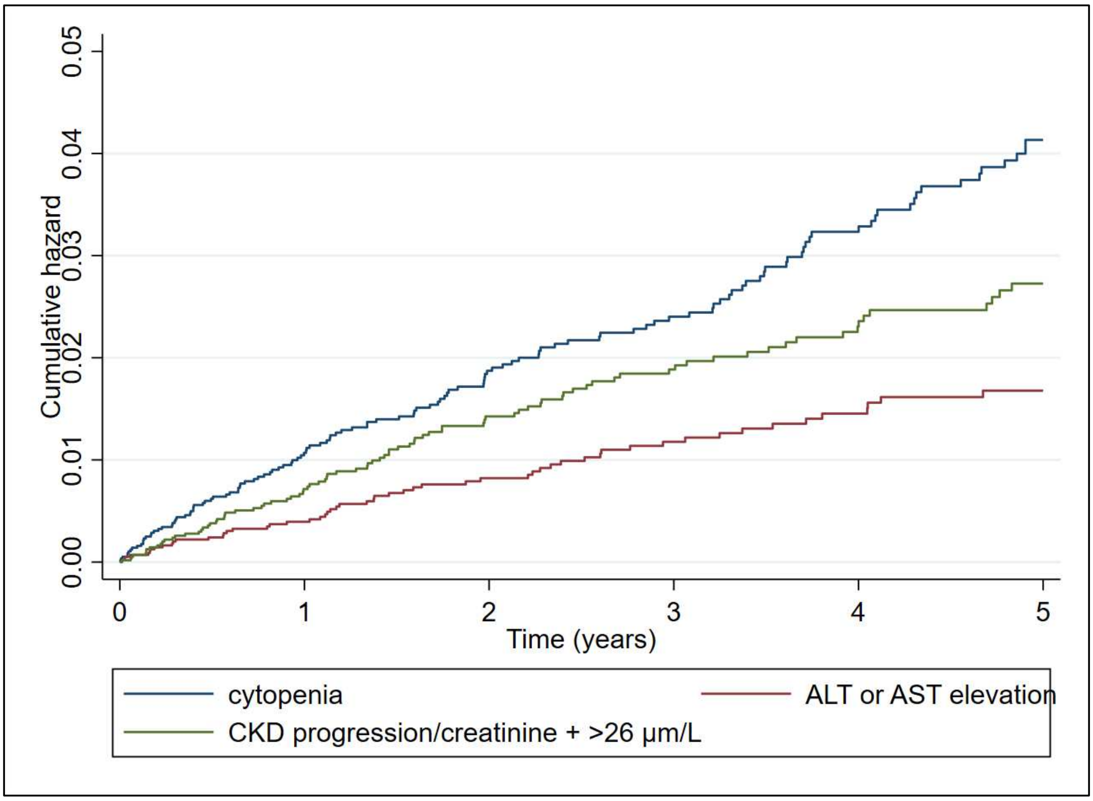

Figure S5: Distribution of predicted risk in the model derivation cohort at 5 years

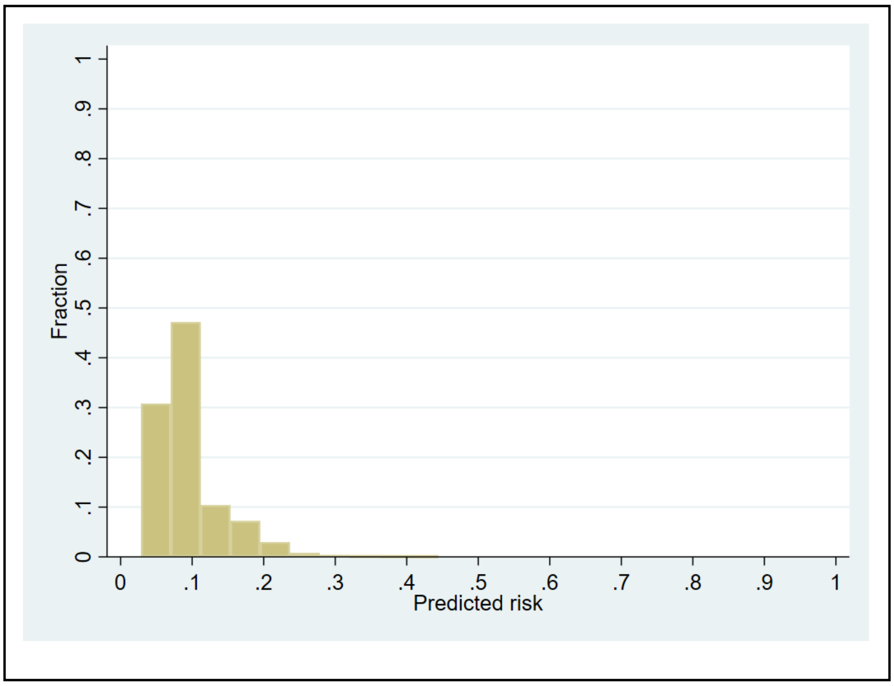

Abhishek A, *et al. RMD Open* 2024; 10:e003980. doi: 10.1136/rmdopen-2023-003980

Figure S6: Calibration of a prognostic model for SSZ discontinuation with abnormal monitoring blood-test results at 5-years in the development cohort[1]

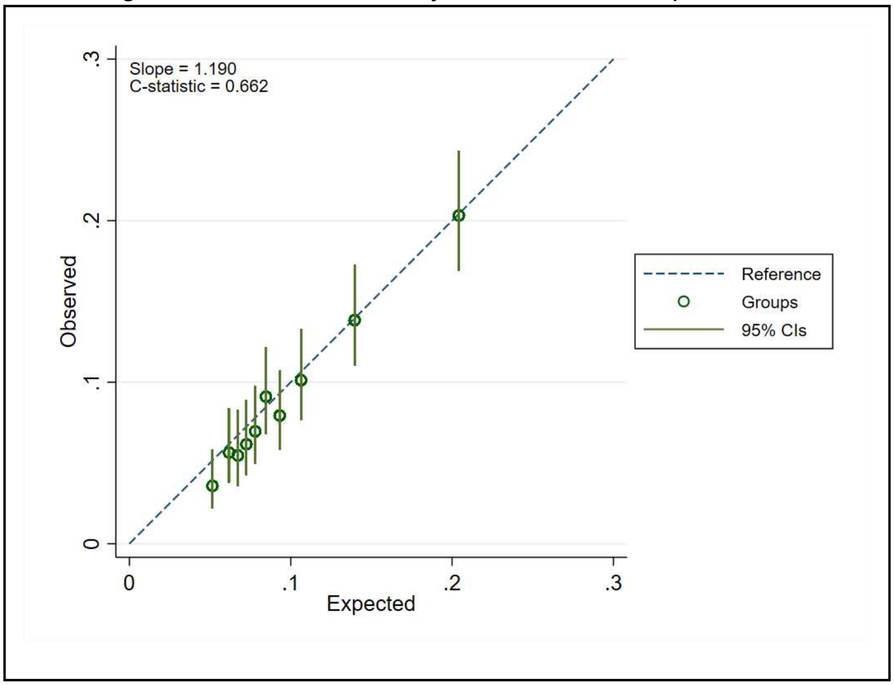

[1]Data from a single imputed dataset; So(t=5) 0.940

Abhishek A, *et al. RMD Open* 2024; 10:e003980. doi: 10.1136/rmdopen-2023-003980

Figure S7: Calibration of a prognostic model for SSZ discontinuation with abnormal monitoring blood-test results at 5-years in the validation cohort[1]

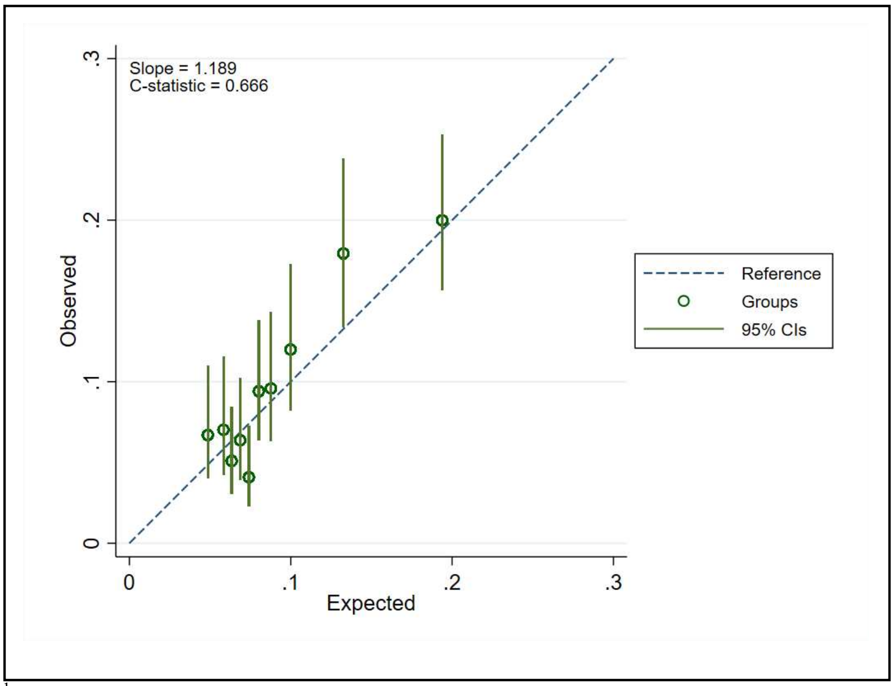

[1]Data from a single imputed dataset; So(t=5) 0.940

Figure S8: Distribution of predicted risk in the model validation cohort at 5 years

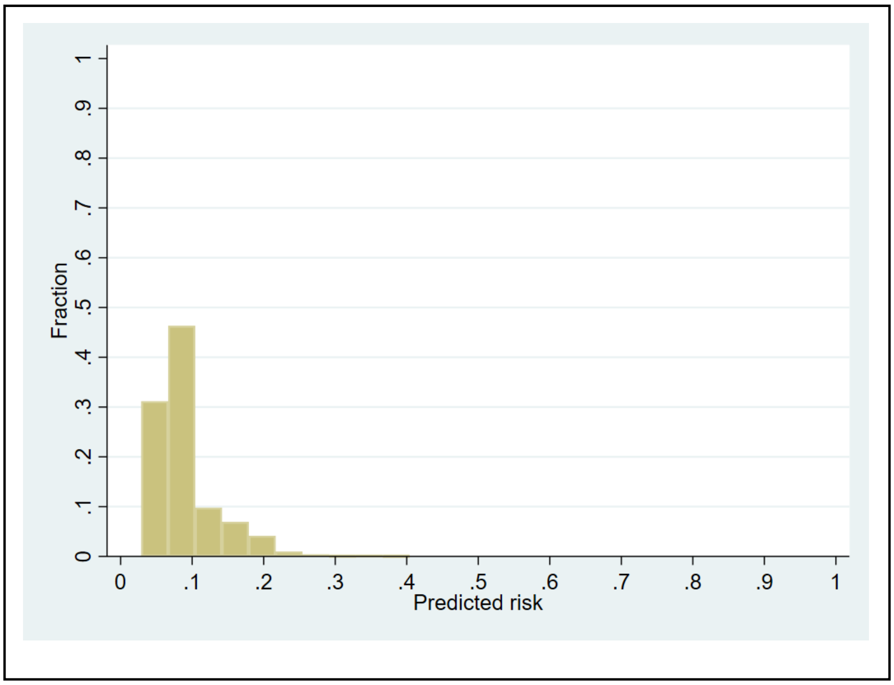

Abhishek A, *et al. RMD Open* 2024; 10:e003980. doi: 10.1136/rmdopen-2023-003980

Figure S9: Calibration of a prognostic model for SSZ discontinuation with abnormal monitoring blood-test results at 1 year in the validation cohort[1]

A: Calibration plot

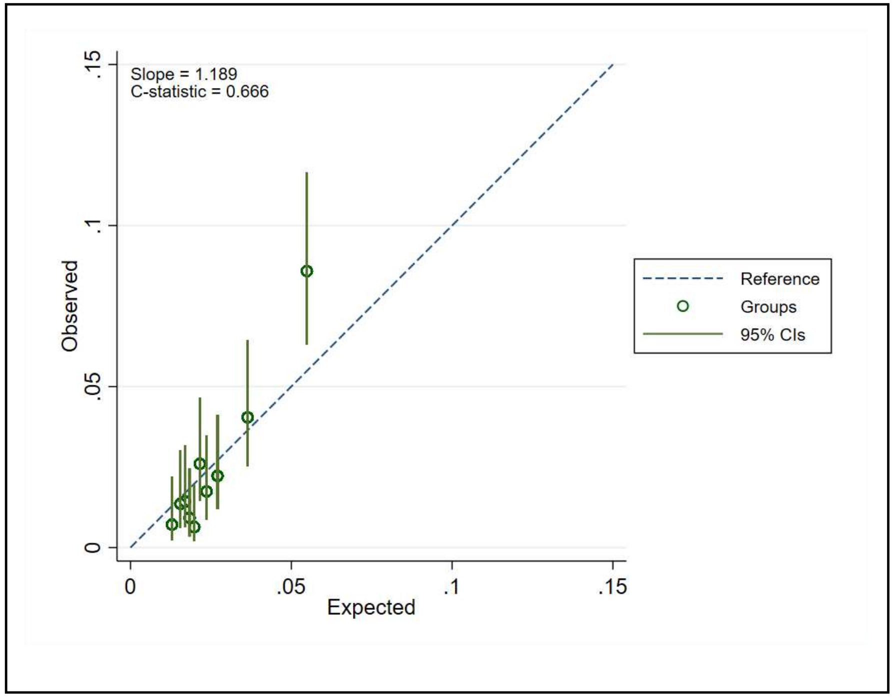

B: Smoothed calibration curve

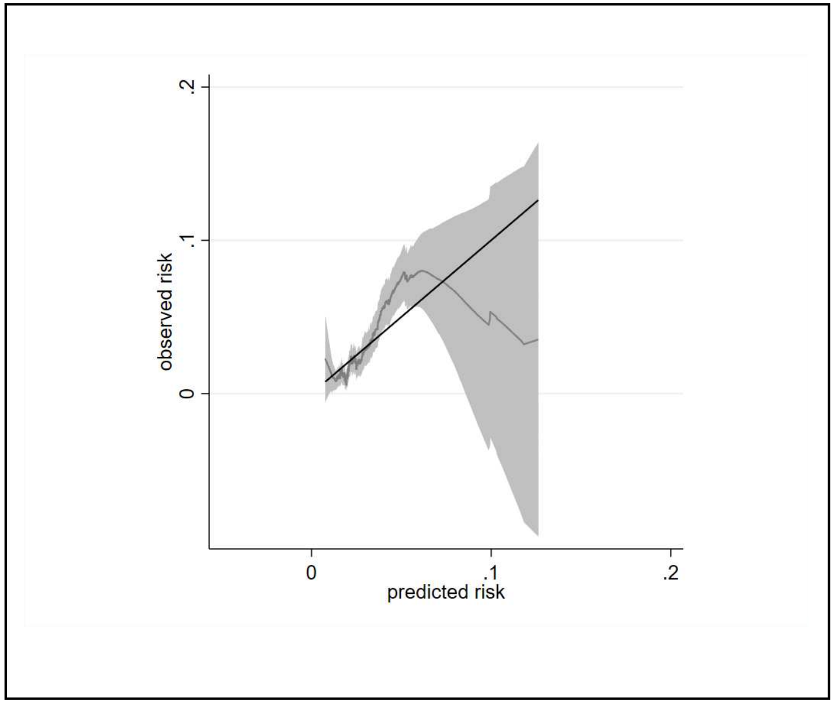

[1]Data from a single imputed dataset was used; $S_o(t_{=1})$ 0.984

Figure S10: Calibration of a prognostic model for SSZ discontinuation with abnormal monitoring blood-test results at 2 years in the validation cohort[1]

A. Calibration plot

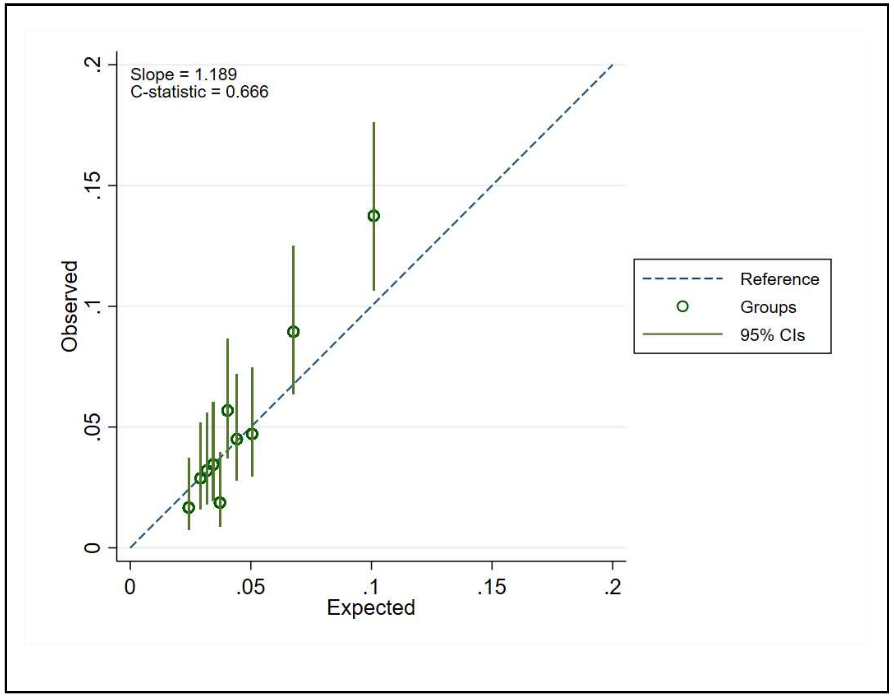

B. Smoothed calibration curve

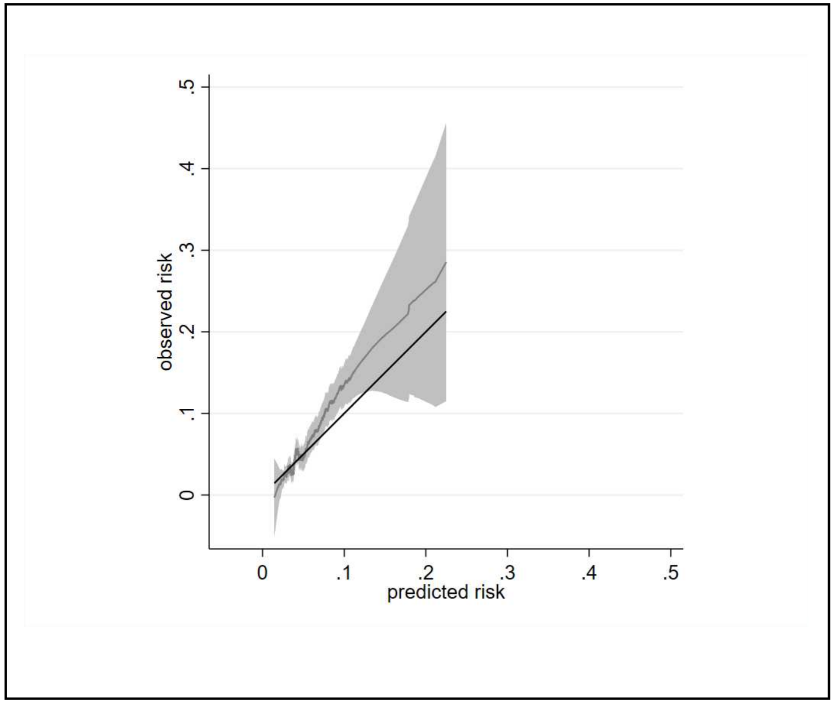

[1]Data from a single imputed dataset was used; $S_o(t_{=2})$ 0.970

Abhishek A, *et al. RMD Open* 2024; 10:e003980. doi: 10.1136/rmdopen-2023-003980

Figure S11: Calibration of a prognostic model for SSZ discontinuation with abnormal monitoring blood-test results at 3 years in the validation cohort[1]

A. Calibration plot

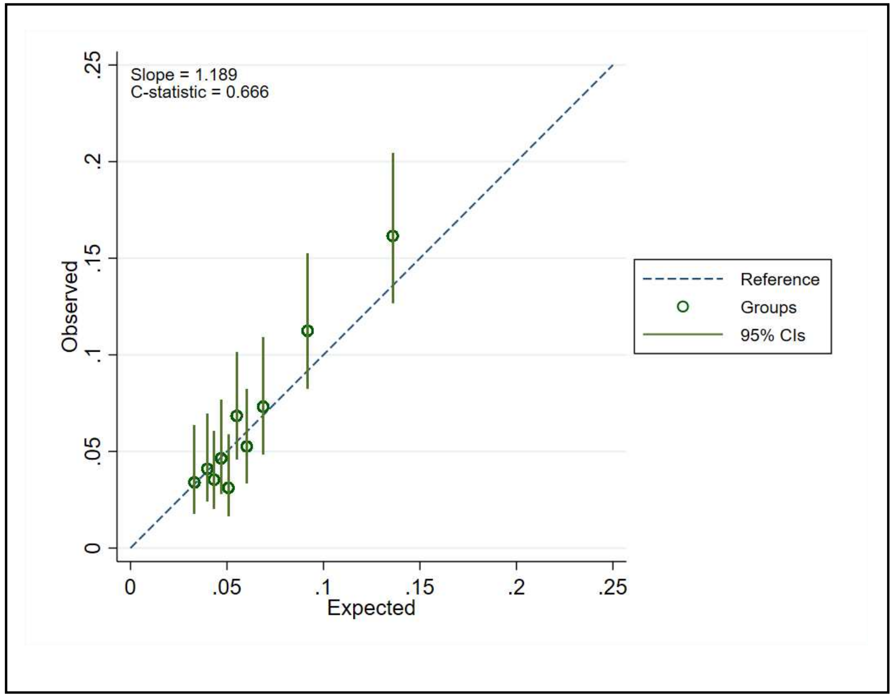

B. Smoothed calibration curve

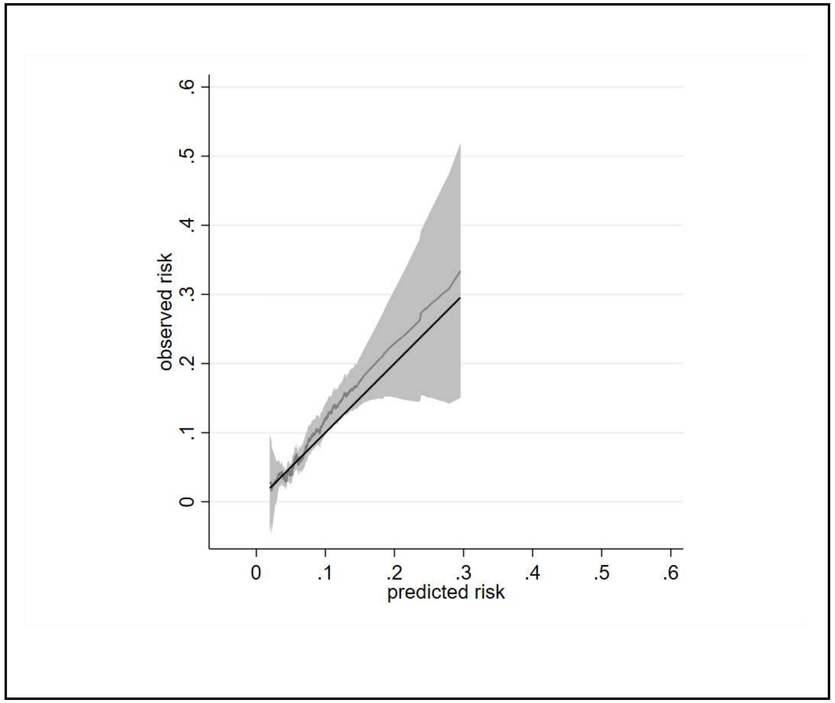

Data from a single imputed dataset was used; $S_o(t_{=3})$ 0.959

Figure S12: Calibration of a prognostic model for SSZ discontinuation with abnormal monitoring blood-test results at 4 years in the validation cohort[1]

A. Calibration plot

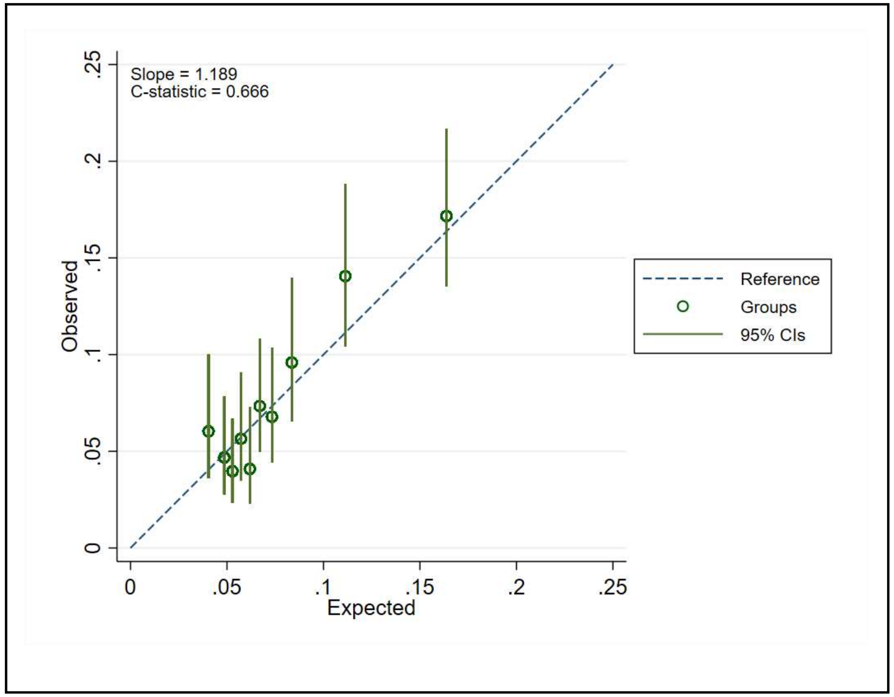

B. Smoothed calibration curve

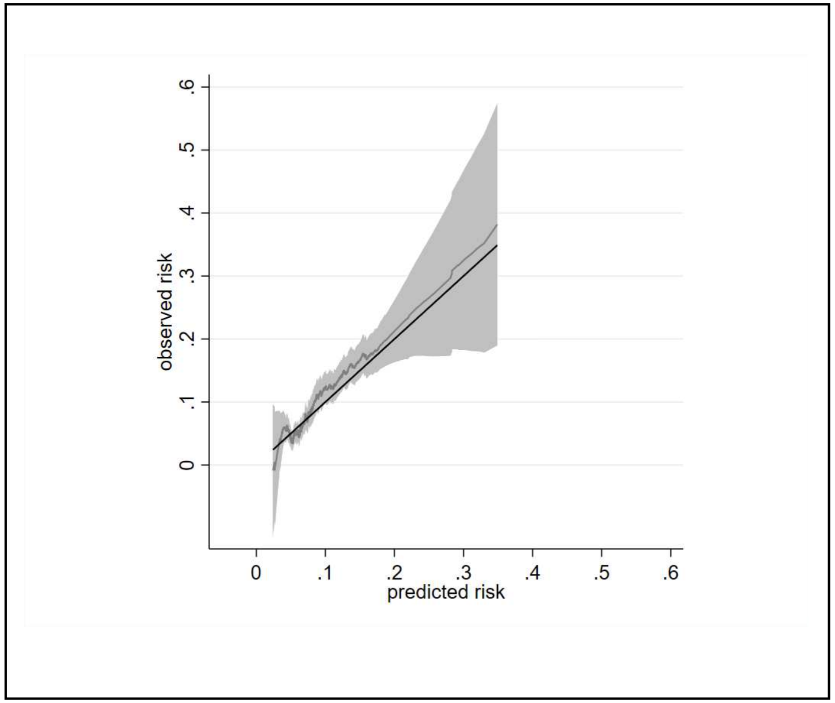

Data from a single imputed dataset was used; $S_o(t_{=4})$ 0.950

Figure S13: Calibration of a prognostic model for SSZ discontinuation with abnormal monitoring blood-test results at 5 years in the validation cohort: stratified according to age.

A. <60 years

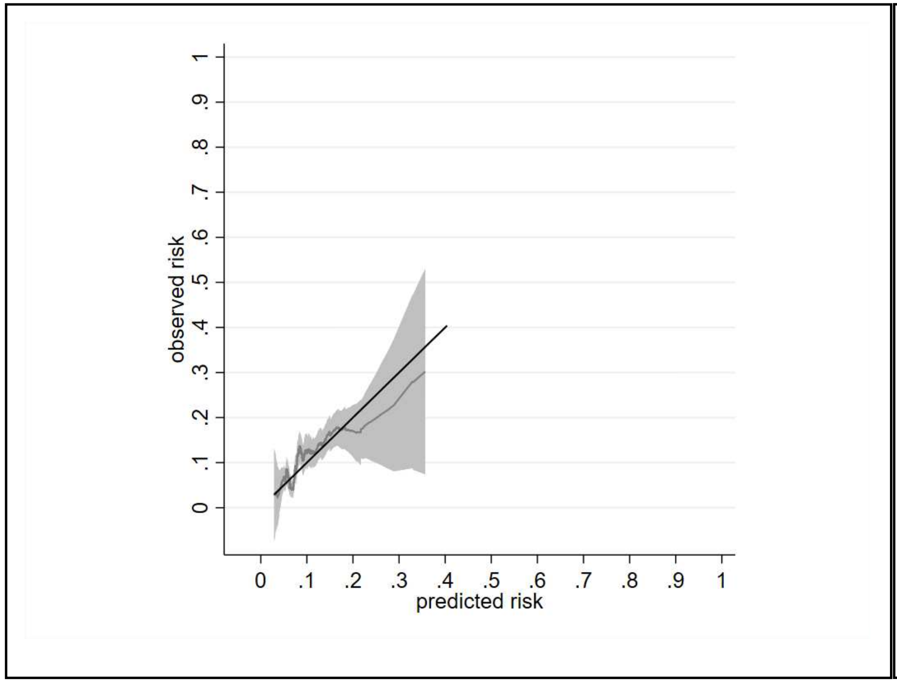

B. ≥ 60 years

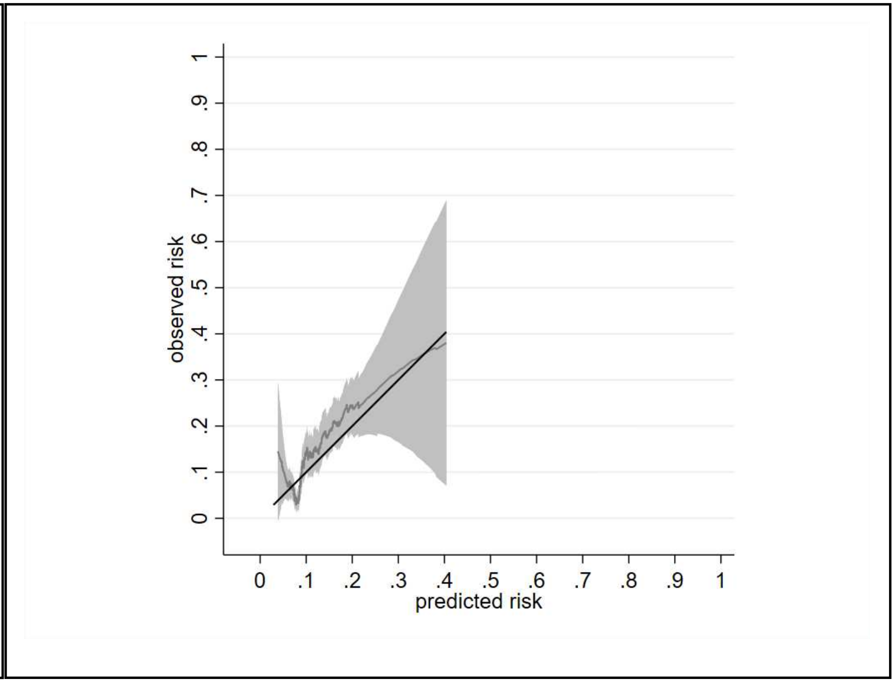

Figure S14: Calibration of a prognostic model for SSZ discontinuation with abnormal monitoring blood-test results at 5 years in the validation cohort: stratified according to disease type.

A: Rheumatoid Arthritis

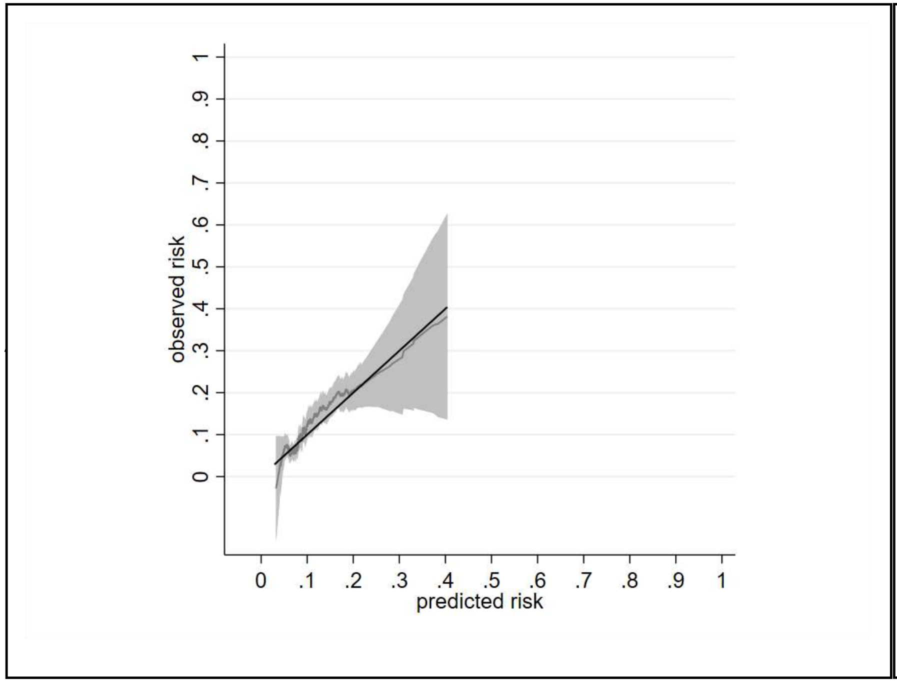

B: other inflammatory conditions

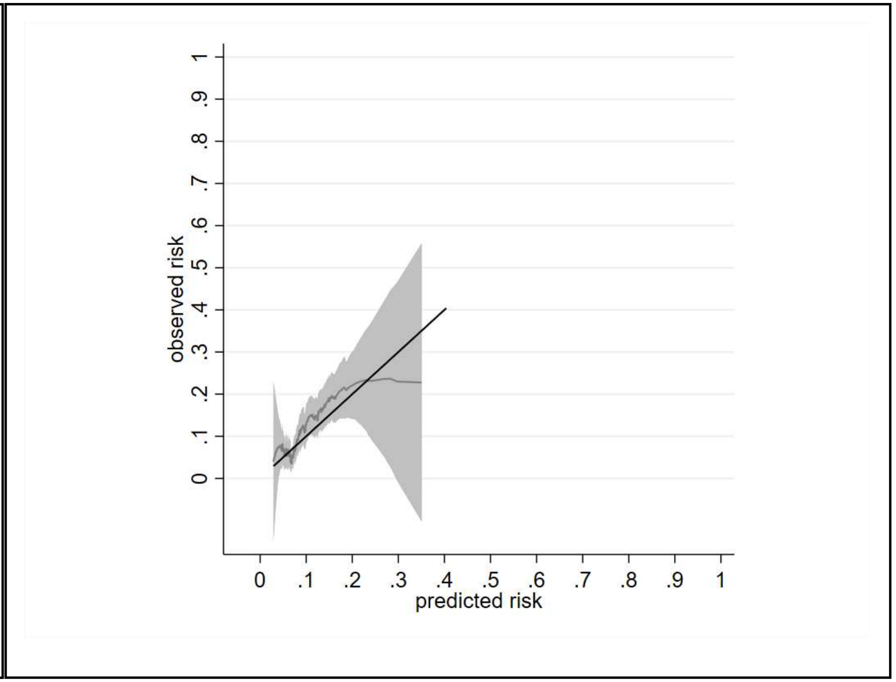

Supplement: Supplementary data [file rmdopen-2023-003980supp001.pdf]
